# Executive Function, Behavioral Self-Regulation, and School Related Well-Being Did Not Mediate the Effect of School-Based Physical Activity on Academic Performance in Numeracy in 10-Year-Old Children. The Active Smarter Kids (ASK) Study

**DOI:** 10.3389/fpsyg.2018.00245

**Published:** 2018-02-28

**Authors:** Katrine N. Aadland, Eivind Aadland, John R. Andersen, Arne Lervåg, Vegard F. Moe, Geir K. Resaland, Yngvar Ommundsen

**Affiliations:** ^1^Faculty of Education, Arts and Sports, Western Norway University of Applied Sciences, Bergen, Norway; ^2^Faculty of Health and Social Sciences, Western Norway University of Applied Sciences, Bergen, Norway; ^3^Department of Education, Faculty of Educational Sciences, University of Oslo, Oslo, Norway; ^4^Department of Coaching and Psychology, Norwegian School of Sport Sciences, Oslo, Norway

**Keywords:** executive function, behavioral self-regulation, school related well-being, elementary school children, structural equation modeling

## Abstract

Inconsistent findings exist for the effect of school-based physical activity interventions on academic performance. The Active Smarter Kids (ASK) study revealed a favorable intervention effect of school-based physical activity on academic performance in numeracy in a subsample of 10-year-old elementary schoolchildren performing poorer at baseline in numeracy. Aiming to explain this finding, we investigated the mediating effects of executive function, behavioral self-regulation, and school related well-being in the relation between the physical activity intervention and child’s performance in numeracy. An ANCOVA model with latent variable structural equation modeling was estimated using data from 360 children (the lower third in academic performance in numeracy at baseline). The model consisted of the three latent factors as mediators; executive function, behavioral self-regulation, and school related well-being. We found no mediating effects of executive function, behavioral self-regulation or school related well-being in the relationship between the ASK intervention and academic performance in numeracy (*p* ≥ 0.256). Our results suggest that the effect of the intervention on performance in numeracy in the present sample is not explained by change in executive function, behavioral self-regulation, or school related well-being. We suggest this finding mainly could be explained by the lack of effect of the intervention on the mediators, which might be due to an insufficient dose of physical activity.

**Trial registration:** Clinicaltrials.gov registry, trial registration number: NCT02132494.

## Introduction

Cluster randomized controlled studies (c-RCT) have shown inconsistent findings regarding the effect of physical activity interventions incorporated in the school curriculum on children’s academic performance (Donnelly and Lambourne, 2011; Beck et al., 2016; Mullender-Wijnsma et al., 2016; Riley et al., 2016; Donnelly et al., 2017). Our group conducted The Active Smarter Kids (ASK) study (Resaland et al., 2015) and found no effect of a 7-month physical activity intervention on academic performance in numeracy, reading, or English (second language) in the overall sample (Resaland et al., 2016). Yet, a favorable effect of the intervention on numeracy was found in children who performed the poorest in numeracy at baseline (the lowest tertile). Because this effect was most likely not a result of an increased physical activity dose, the effect might stem from how physical activity was integrated in the learning activities. This hypothesis is consistent with studies showing a favorable effect of physical activity integrated in academic lessons (Bartholomew and Jowers, 2011; Donnelly and Lambourne, 2011; Norris et al., 2015; Beck et al., 2016; Mullender-Wijnsma et al., 2016). A closer examination of possible mediators of this effect may increase our understanding of how physical activity at school can improve academic performance in those children most in need. Hence, we aimed to explore the mediating role of executive function, behavioral self-regulation, and school related well-being.

Executive function can be defined as “the cognitive processes necessary for goal-directed cognition and behavior” (Best, 2010, p. 331). Core executive functions involve inhibition and interference control, working memory, and cognitive flexibility (Miyake et al., 2000; Diamond, 2013). The importance of executive function for academic performance is well documented (Best et al., 2011; Cantin et al., 2016). Furthermore, promising evidence exists for a positive effect of physical activity on executive function (Donnelly et al., 2016; Vazou et al., 2016). Possible pathways in which physical activity can affect executive function are through physiological responses in the brain, and through cognitive and motor challenging activities that improve cognitive skills that can be transferred to executive function tasks (Best, 2010). Supporting these pathways, studies have shown favorable effects of physical activity on both brain function and structure in children (Chaddock et al., 2011; Khan and Hillman, 2014). Furthermore, physical activity interventions with increased cognitive engagement, through either enhanced cognitive demands and/or execution of complex motor skills, have shown superior effects on executive functions compared to physical activity without this enhancement (Pesce et al., 2013; Crova et al., 2014; Schmidt et al., 2015; Koutsandreou et al., 2016). In a school setting, incorporating physical activity in academic lessons may prove a particularly appealing way of enhancing the cognitive demands of physical activity, as it preserves the scheduled time to academic teaching. In support of this approach, Vazou and Smiley-Oyen (2014) revealed larger improvements in response time in a task emphasizing inhibition control following an acute 10 min bout of physical activity integrated with math practice, compared to seated math practice.

Besides the important contribution of executive function to academic performance, behavioral self-regulation skills are important as they enables children to adapt successfully to classroom demands and engage in learning opportunities (McClelland and Cameron, 2012). Behavioral self-regulation requires coordination of executive functions along with motor and verbal functions, and includes behavioral skills such as paying attention, following instructions, and inhibiting inappropriate actions (McClelland et al., 2007). Hence, measures of context specific behavioral self-regulation in the classroom through teacher reports add ecological validity to tests of executive function.

Behavioral self-regulation is important for academic performance (McClelland and Cameron, 2012), and several studies have revealed predictive effects of behavioral self-regulation on academic performance (von Suchodoletz et al., 2013; Gestsdottir et al., 2014; Schmitt et al., 2014; Bryce et al., 2015). Few studies, however, have investigated the effect of physical activity on behavioral self-regulation in elementary school children (Lubans et al., 2016). Some studies have investigated the effect of incorporating physical activity during the school day on attention and on-task behavior (Mahar et al., 2006; Bartholomew and Jowers, 2011; Kibbe et al., 2011; Mahar, 2011; Carlson et al., 2015; Riley et al., 2016). Carlson et al. (2015) found that teacher implemented classroom physical activity break of 10 min was positively related to better on-task and attentive behavior in the classroom. Riley et al. (2016) revealed a positive effect of an intervention incorporating physical activity in the pre-existing mathematics program (3 × 60 min/week) over 6 weeks on on-task behavior (observation) during the mathematics lessons. Other studies, have observed increased time on-task behavior after combining physical activity and academic lessons (Mahar et al., 2006; Bartholomew and Jowers, 2011; Mahar, 2011) with greatest effect in those children exhibiting least on-task behavior before the intervention.

The effect of a physical activity intervention on academic performance in numeracy might also be affected through a psychosocial mechanism in which school related well-being is triggered (Bailey, 2016). Physical activity provides a natural setting for development of friendship and peer relationships, social identities, and belonging, all of which are important nutrient’s for children’s social engagement or well-being at school (Bailey, 2016). Supporting this link, Haapala et al. (2014) found that physical activity during recess was positively associated with peer relationships at school, relatedness to school, and school climate. In turn, research has consistently shown that social belonging and peer acceptance relate positively to pursuit of goals to learn, interest in school, and perceived academic competence and academic accomplishments (Wentzel, 2017). Furthermore, academic peer popularity is observed to mediate the relationship between self-regulation (attentional control) and academic performance in mathematics in elementary schoolchildren (Sanchez-Perez et al., 2015). As examples of activities used in the ASK intervention, socially reinforcing co-operative group based physical activities have been shown to stimulate peer relations and satisfaction of the need for social relatedness, which might enhance well-being at school and intrinsic regulation of school motivation (Ryan and Moller, 2017).

**FIGURE 1:**
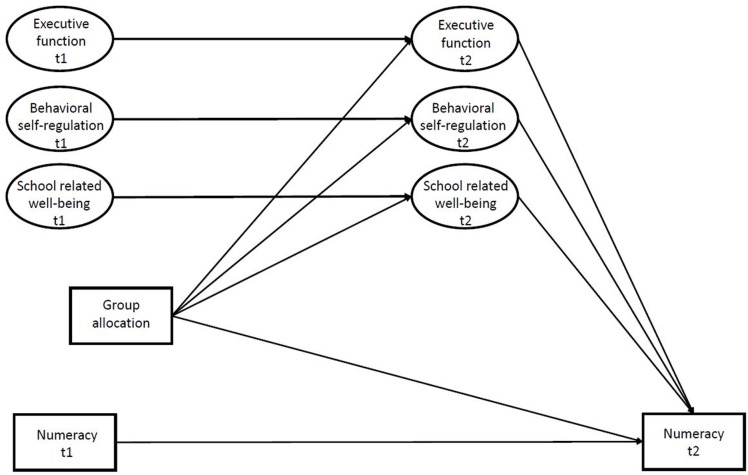
The hypothesized model. t1 = baseline; t2 = follow-up; group allocation = intervention/control.

Against this background, we aimed to investigate if improved executive function, behavioral self-regulation, and school related well-being mediated the observed effect of the ASK intervention on performance in numeracy for those performing poorest in numeracy at baseline. We hypothesized that the effect of the intervention on performance in numeracy, worked through improvement in executive function, behavioral self-regulation, and school related well-being (**Figure 1**).

## Materials and Methods

### Design and Participants

The ASK study is a parallel group (intervention group vs. control group, 1:1 ratio) cluster-randomized controlled trial conducted in Sogn og Fjordane county, Norway, between August 2014 and June 2015 (Resaland et al., 2015). Randomization was performed by a neutral third part (Centre for Clinical Research, Haukeland University Hospital, Norway) and the unit of randomization was the participating schools. The procedures and methods used in the ASK trial conform to the ethical guidelines defined by the World Medical Association’s Declaration of Helsinki and its subsequent revisions (WMA, 2013). The study protocol was approved by The Regional Committees for Medical Research Ethics (REC southeast). We obtained written informed consent from each child’s parent(s)/guardian(s) prior to all testing.

**FIGURE 2:**
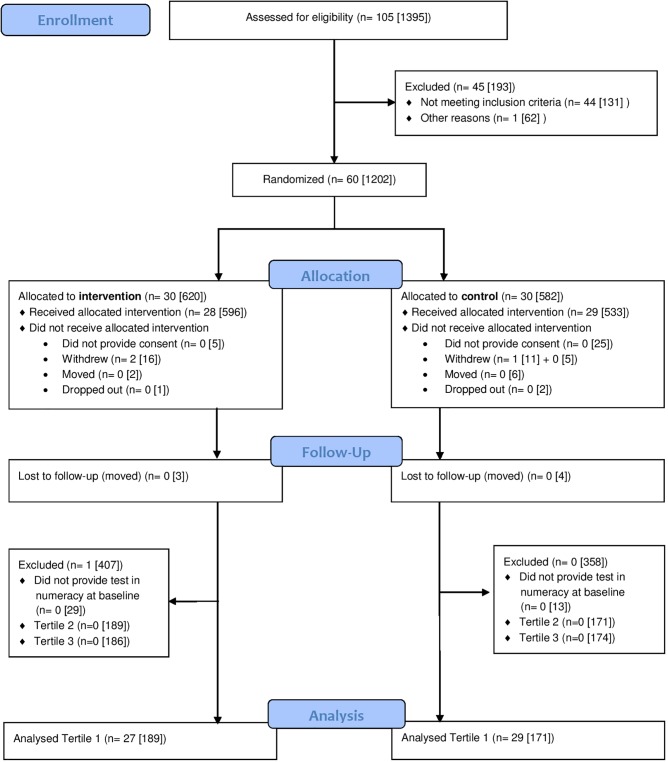
Flow diagram of the included children (*n* = schools [children]).

We invited all 60 schools in the county that fulfilled the inclusion criteria of at least seven fifth-grade children enrolled. All schools, encompassing 1202 fifth-grade children, agreed to participate (**Figure 2**). Three schools (one control school and two intervention schools) from the same municipality resigned after randomization. In total, 1145 (82.1 % of the population of 10-year-olds in the county) of the 1175 invited children from 57 schools agreed to participate. The present study includes children in the lowest tertile of academic performance in numeracy at baseline. The study was registered in the Clinicaltrials.gov registry [trial registration number: NCT02132494] prior to commencement.

### Intervention

The ASK intervention was a part of the mandatory school curriculum for all children attending the intervention schools. It was led by the classroom teachers and consisted of three components (165 min/week); (1) physically active educational lessons, mostly performed outdoor in the school yard (3 × 30 min/week) in the subjects Norwegian, Mathematics and English, (2) physical activity breaks during classroom lessons (5 min/school-day) and (3) physical activity homework (10 min/school-day). The research group developed the intervention in collaboration with teachers at the intervention schools. Our mutual aim was to create a number of varied physical activity activities that should be carried out in small groups and which encouraged an inclusive and joyful learning environment (examples of physical activity activities^1^). Two of the three intervention components, that is, the physical activity educational lessons and the physical activity homework, incorporated academic learning tasks in the physical activity (e.g., rope jumping while spelling English vocabulary words), thus adding cognitive load to the activity. Approximately 25% of the daily physical activity in the ASK intervention was intended to be of vigorous intensity. This was defined as “children would be sweating and out of breath.” Intervention schools were provided with equipment for use in the intervention activities and the children received a skipping rope and a tennis ball for physical activity homework. To ensure that the teachers were empowered, supported, and qualified to deliver the ASK intervention, we conducted three instructional seminars ahead of the intervention start for the teachers at the intervention schools. Further, we provided two regional refresher sessions during the intervention period. Finally, we provided teachers in intervention schools with e-mail- and telephone-support, as well as password-protected homepage^2^ that supplied teachers with information, videos, and physical activity lessons.

Children from both intervention schools and control schools participated in curriculum-prescribed 90 min/week of physical education and 45 min/week of physical activity (total 135 min/week). It was specified to the control schools that they should carry out the amount of physical activity and physical education that they would have done regardless of the ASK study.

### Measures

#### Anthropometry, Pubertal Status, and Socio Economic Status

Body mass (weight; 0.1 kg) was measured using an electronic scale (Seca 899, SECA GmbH, Hamburg, Germany) with children wearing light clothing. Stature (height; 0.1 cm) was measured with portable Seca 217 (SECA GmbH, Hamburg, Germany). We calculated body mass index (kg m^-2^) as weight (kg) divided by the height squared (m^2^). Body fat was measured using four skinfold thickness sites (biceps, triceps, subscapular, and suprailiac) using a Harpenden skinfold caliper (Bull; British Indicators Ltd., West Sussex, England) according to the criteria described by Lohman et al. (1991). The Harpenden skinfold caliper has been tested for validity and reliability in children (Yeung and Hui, 2010). Children self-assessed their pubertal stage with the Tanner method (Tanner, 1962) using a scale of colored images proposed by Carel and Leger (2008). We used pubic hair for both sexes and breast and genital development for girls and boys, respectively. Socio economic status (the highest education level obtained by the mother or father) (Sirin, 2005) was reported by the parents or guardians.

#### Mediators

##### Executive function

We measured key executive functions identified by Miyake et al. (2000); inhibition, working memory, and cognitive flexibility, by using four pen and paper tests. We assessed inhibition with the Stroop Color and Word Test (Stroop CW) (Golden, 1978). To assess cognitive flexibility, we used a semantic Verbal Fluency test (Spreen and Strauss, 1998), and The Trail Making Test (TMT) (Spreen and Strauss, 1998; Lezak et al., 2012). Finally, we used a Digit Span test (Digits Forward and Backward) from the Wechsler Intelligence Scale for Children, fourth ed. (WISC-IV) to assess working memory (Lezak et al., 2012). All tests of executive function are validated for use in children (Wechsler, 2003; Reitan and Wolfson, 2004; Ardila et al., 2006; Peru et al., 2006), and shows good reliability (Neyens and Aldenkamp, 1997; Spreen and Strauss, 1998; Homack and Riccio, 2004; Lezak et al., 2012). For a more thorough description of the executive functions tests, see Aadland et al. (2017a). We treated executive function as one latent factor including the Color-Word task of the Stroop Test (Stroop CW), Verbal Fluency total, the Digit Backward of WISC-IV (WISC-IV backward), and the Trail Making Test part B (TMT-b).

##### Behavioral self-regulation

We assessed behavioral self-regulation with the 10 items from the Child Behavior Rating Scale (CBRS) (Bronson et al., 1990), identified to describe child behavioral self-regulation in a classroom setting (Gestsdottir et al., 2014). Teachers were asked to rate children’s classroom behavior on a five point Likert scale ranging from 1 (never) to 5 (always) to indicate how frequently a given behavior occurred. The CBRS is a reliable and valid tool that has been used in multiple studies in Western countries (Lim et al., 2010; von Suchodoletz et al., 2013). Cronbach’s alpha for the CBRS for the present sample was 0.95 both at baseline and follow-up. A mean score of the CBRS was used for descriptive statistics, and a latent behavioral self-regulation factor was used in the mediation analysis.

##### School related well-being

Quality of life was assessed by self-reporting using the Kidscreen-27 questionnaire (Ravens-Sieberer et al., 2007), which consists of 27 items covering the following five quality of life dimensions: (1) physical well-being (5 items); (2) psychological well-being (7 items); (3) parent/guardians relations & autonomy (7 items); (4) social support & peers (4 items); and (5) school environment (4 items). The Kidscreen-27 questionnaire has been validated in Norwegian children (Haraldstad et al., 2011; Andersen et al., 2015). Cronbach’s alpha for the school environment dimension for the present sample was 0.78 at baseline and 0.85 at follow-up. For descriptive statistics, *T*-scores were obtained according to the developer’s manual, where a mean of 50 and a standard deviation (SD) of 10 define normality for children in Europe. Higher score indicate better school related well-being. For the mediation analysis, we composed a latent school related well-being factor from the four items concerning school environment (Questions: (1) “Have you been happy at school?” (2) “Have you got on well at school?” (3) “Have you been able to pay attention?” (4) “Have you got along well with your teachers?”).

#### Outcome Measure

##### Academic performance in numeracy

We measured academic performance in numeracy using a specific standardized Norwegian National test designed and administered by the Norwegian Directorate for Education and Training (NDET). The tests have shown evidence of good validity and reliability by NDET and are aligned with the competencies demanded from all schools by the national curriculum (Resaland et al., 2015). The score is reported as standardized points, with a mean of 50 and a standard deviation of 10.

### Power Calculations

The ASK study was designed to detect an effect size (Cohen’s *d*) of 0.35 between the two groups for change in academic performance (main outcome). The effect size was based on findings from previous studies (Sibley and Etnier, 2003). An intra-class correlation (ICC) of 0.15 [observed clustering of academic performance during the previous school year (2013–2014)] was applied to account for the cluster-randomized design, leading to a design effect of 4.54. For further details, see Resaland et al. (2015).

### Statistics

All study variables were examined for distributional properties, of which TMT-b was transformed and other variables kept in theirs original form. We excluded all values exceeding five standard deviations from the mean.

Children’s characteristics are provided as means and standard deviations (SD) or frequencies. A linear mixed model including school as a random effect was used to examine differences between children in tertiles of numeracy. The descriptive analyses were conducted with SPSS software, version 23.0 (IBM SPSS Statistics for Windows, : IBM Corp., Armonk, NY, United States).

Structural equation modeling (SEM), with full information maximum likelihood estimation (FIML) was used to examine bivariate correlations and the mediation model. The analyses were implemented through the Mplus program, version 7.4 (Muthén and Muthén, 1998–2015). To estimate the mediating effects in our pretest–posttest control group design, we used the ANCOVA model as recommended and explained by Valente and MacKinnon (2017). To account for children nested within schools, the complex method with robust maximum likelihood (MLR) estimator was used in all analyses. To reduce the complexity of the mediation model, we made five parcels from the 10 items in the CBRS. The parcels were composed by a balanced approach described in Little (2013), and as we had two measurement time points we used the average loading across the time points to rank the items.

Multiple fit indices in addition to the chi-square test statistic were used to assess model fit; the Comparative Fit Index (CFI), the Root Mean Squared Error of Approximation (RMSEA), and the Standardized Root Mean Square Residual (SRMR). We used a non-significant χ^2^ and the cutoff recommendations of CFI > 0.95, and RMSEA and SRMR < 0.05 as indications of good model fit to the data (Geiser, 2013).

Measurement invariance (metric and scalar) was tested across time and group for the latent factors of executive function, behavioral self-regulation, and school related well-being. Criteria used to test differences between nested models (configural, metric, and scalar) were ΔCFI of -0.010, ΔRMSEA of 0.015, and ΔSRMR of 0.010 (for scalar) as recommended by Chen (2007) for sample size *N* > 300. A *p*-value ≤ 0.05 was used to indicate statistical significance in all analyses.

## Results

The characteristics of the children included in the present study (tertile 1), as well as the other children constituting the remaining ones in the ASK study (tertile 2 and 3) are shown in **Table 1**. Children in tertile 1 had significantly higher skinfold thickness than children in tertile 3 (*p* = 0.008), but no statistical differences were observed between children in tertile 1 versus tertile 2 and 3 children for BMI, pubertal status, or socio economic status (*p* > 0.127). Children in tertile 1 performed significantly poorer on all tests of executive function and academic performance in numeracy, and had significantly lower score on measures of behavioral self-regulation (CBRS) and school related well-being (Kidscreen, school environment) than children in tertile 2 and 3. Bivariate correlations between the mediators and the outcome are shown in **Table 2**.

**Table 1:** Baseline characteristics of the children as means [standard deviations (SD)] or frequencies of the present sample (tertile 1 = lowest performance in numeracy) vs. the other children in the total ASK study sample (tertile 2 and 3).

	Tertile 1		Tertile 2		Tertile 3	
Variable	*n*	*M (SD)/%*	*n*	*M (SD)/%*	*n*	*M (SD)/%*
Age (years)	360	10.2 (0.31)	360	10.2 (0.28)	360	10.2 (0.27)
Sex (%)						
Girls	177	49.2	194	53.9	147	40.8
Boys	183	50.8	166	50.8	213	59.2
BMI	355	18.3 (3.3)	348	18.1 (3.15)	348	17.7 (2.4)
Skinfold thickness (mm)	352	52.4 (28.5)	347	51.6 (27.6)	345	46.1 (23.1)^∗∗^
Pubertal stage (Tanner) (%)						
Stage 1	98	27.2	103	28.6	94	26.1
Stage 2	210	58.3	201	55.8	216	60.0
Stage 3–5	43	12.0	42	11.7	35	9.7
Socio economic status (%)						
≤Upper secondary school	117	32.5	105	29.2	119	33.1
<4 years of university	104	29.9	98	27.2	136	37.8
≥4 years of university	112	31.1	100	27.8	130	36.1
Executive function						
Stroop CW (*n*)	352	23.6 (5.2)	355	26.1 (5.5)^∗∗∗^	346	28.1 (6.1)^∗∗∗^
Verbal Fluency (*n*)	353	14.8 (4.3)	357	16.4 (4.4)^∗∗∗^	348	17.2 (4.7)^∗∗∗^
WISC-IV b (*n*)	353	5.7 (1.1)	355	6.3 (1.2)^∗∗∗^	350	6.7 (1.4)^∗∗∗^
TMT-b (s)	331	147(59)	345	115 (34)^∗∗∗^	351	102 (33)^∗∗∗^
Behavioral self-regulation	334	3.4 (0.8)	333	4.0 (0.7)^∗∗∗^	317	4.2 (0.6)^∗∗∗^
School related well-being (*T*-score)	298	51.9 (10.2)	314	54.0 (9.6)^∗^	325	54.9 (9.1)^∗∗∗^
Academic performance						
Numeracy	360	40.7 (3.9)	360	51.1 (2.6)^∗∗∗^	360	62.0 (4.7)^∗∗∗^

*BMI, body mass index; Stroop CW, Stroop Color Word; WISC-IV b, Wechsler Intelligence Scale for Children, fourth edition, backward digit span; TMT-b, Trail Making Test part B; ^∗^p < 0.05 compared to tertile 1; ^∗∗^p < 0.01; ^∗∗∗^p < 0.001 compared to tertile 1.*

**Table 2:** Estimated correlation matrix for the mediators and the outcome at baseline (above the diagonal line) and at follow-up (below the diagonal line).

	Executive function	Behavioral self-regulation	School related well-being	Numeracy
Executive function	–	**0.40**	0.10	**0.34**
Behavioral self-regulation	**0.37**	–	**0.24**	**0.20**
School related well-being	0.09	**0.25**	–	**0.21**
Numeracy	**0.40**	**0.19**	0.06	–

*Significant correlations (p < 0.05) are shown in bold.*

The confirmatory factor analysis (CFA) for the three latent variables showed scalar invariance across time and group with ΔCFI, ΔRMSEA, and ΔSRMR below suggested criteria comparing the scalar against the configural model [Δχ^2^*(*Δ*df*) = 28.04 (*18*) (*p* = 0.245), ΔCFI = -0.001, ΔRMSEA = 0.001, ΔSRMR = 0.009, and Δχ^2^(Δ*df*) = 61.02 (*36*) (*p* = 0.006), ΔCFI = -0.006, ΔRMSEA = 0.002 ΔSRMR = 0.011 across time and group, respectively]. Factor loadings for the observed variables in the three latent variables are presented in Supplementary Table S1.

### The Mediation Model

**FIGURE 3:**
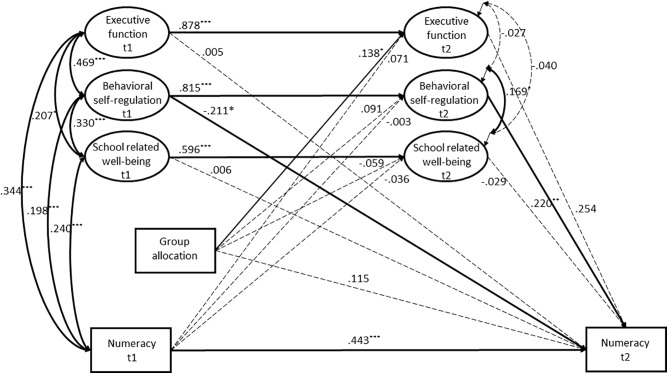
The mediation model. All path coefficients are reported as standardized β-estimates. Significant paths are in bold lines. t1 = baseline, t2 = follow-up. ^∗^*p* ≤ 0.05, ^∗∗^*p* ≤ 0.01, ^∗∗∗^*p* ≤ 0.001.

Our results revealed that neither executive function, behavioral self-regulation, nor school related well-being mediated the effect of the intervention on academic performance in numeracy (**Figure 3**). Standardized β-coefficients (unstandardized *p*-values) for the indirect effects were 0.035 (*p* = 0.479) for executive function, 0.020 (*p* = 0.256) for behavioral self-regulation, and 0.002 (*p* = 0.714) for school related well-being. The mediation model had good fit [χ^2^(*df*) = 433.178 (*307*) (*p* < 0.001), RMSEA = 0.034 (90% CI, 0.026,0.041), CFI = 0.976, and SRMR = 0.049].

## Discussion

In the present study, we aimed to investigate mediators for the effect of the ASK school-based physical activity intervention on academic performance in numeracy for the poorest performing children in numeracy at baseline. Contrary to our hypothesis, we found that neither executive function, behavioral self-regulation, nor school related well-being mediated the effect.

Compared to their peers, the children included in the present study scored significantly lower on all mediators at baseline. Hence, the potential for the intervention to affect these mediators may have been larger for this group of children. In support of this hypothesis, we found an effect of the intervention on executive function in the present study, while this was not evident in a previous study including the total ASK study sample (Aadland et al., 2017c). The present finding is in line with previous studies, where children most behind on executive function are shown to benefit the most from any interventions to increase executive function (Diamond, 2013). However, the intervention did not affect behavioral self-regulation or school related well-being.

### The Role of Executive Function

The effect of the intervention on executive function despite no difference in objectively measured physical activity between the intervention and control schoolchildren, suggests that the content of the intervention was of importance. The prevailing literature supports the value of the cognitive demands inherent in the physical activities in order to affect executive function (Best, 2010; Pesce, 2012; Diamond, 2015; Tomporowski et al., 2015). The ASK intervention might have enhanced the cognitive demands of the physical activity in several ways. First, the integration of academic learning tasks while being physically active likely increased the challenge on executive functions, as these functions are important for solving for example academic problem-solving tasks in mathematics (Bull and Scerif, 2001; Cantin et al., 2016). To our knowledge, no previous study have investigated the effect on executive function using physical activity integrated in mathematics lessons in a sample of children performing lower in numeracy. In general samples of elementary schoolchildren, no effects of physical activity integrated in academic lessons on executive function have been observed (Beck et al., 2016; de Greeff et al., 2016). This might indicate that children with lower math competence take unique advantage of getting involved in academic learning tasks while being physically active.

Second, the greater part of the intervention activities was organized as group activities, which require use of strategic and goal-directed behavior through social interactions in an environment that constantly change (Best, 2010). Executive function skills acquired in these activities may transfer to other executive function tasks. Supporting this line of reasoning, Schmidt et al. (2015) observed only intervention effects on executive function following a team-game intervention, and not following an aerobic exercise intervention, although both interventions increased aerobic fitness equally.

There is also a possibility that challenging features of the activities in terms of motor coordination helped make the activities more cognitively demanding. For example, all children in the intervention group received a tennis ball for the homework, which may have resulted in enhanced practice of aiming and catching skills that previously have been shown to act as a mediator in the effect of a physical activity intervention on executive function (Pesce et al., 2016). Furthermore, the daily 5 min physical activity breaks during the academic lessons in the classroom were often coordinative demanding dancing activities (e.g., Just Dance), possibly targeting the same mechanisms as studied by Budde et al. (2008), where coordinative exercise affected attention and concentration. Supporting evidence for the relevance of coordinative demands of chronic physical activity on executive functions have been observed previously (Crova et al., 2014; Koutsandreou et al., 2016).

The observed effect on executive function only in this subsample of the ASK study, could well also be attributed to the fact that the intervention optimally challenged these children in terms of the motor capabilities, as addressed by Pesce et al. (2013). Finally, the effect of the intervention on executive function might also be owing to the fact that the included children had higher body fat than the other children (however, only significantly higher than the best performing children in numeracy). Studies have suggested that overweight children benefit more from physical activity to improve executive function (Davis et al., 2011; Crova et al., 2014).

Contrary to the literature showing a consistent relationship between executive function and academic performance (Best et al., 2011; Cantin et al., 2016), we found no relationship between change in executive function and change in academic performance. However, it represents a conservative design to investigate change in the mediator on change in the outcome, as in the current case. In order to capture the effects of change in the executive function on change in numeracy, a longer follow-up period might be necessary. For example, we have previously observed a significant relation between executive function at baseline and change in numeracy (Aadland et al., 2017b).

### The Role of Behavioral Self-Regulation

We found no effect of the intervention on behavioral self-regulation. This stands in contrast to the effect on executive function and results from previous studies that have observed increased time on-task during or following physically active academic lessons (Bartholomew and Jowers, 2011; Riley et al., 2016) and short physical activity bouts during academic lessons with or without academic instruction (Mahar et al., 2006; Kibbe et al., 2011; Carlson et al., 2015). An explanation for this discrepant finding might be that these previous studies also increased physical activity levels, likely reflecting the importance of the physical activity dose on behavioral self-regulation. More specifically, our physical activity breaks of 5 min during academic lessons were shorter than previous studies reporting effects on on-task resulting from 10 min bouts of physical activity (Mahar et al., 2006; Kibbe et al., 2011; Carlson et al., 2015). Studies that have revealed effects of acute physical activity on arousal and attention have also used bouts of longer durations (Budde et al., 2008; Janssen et al., 2014a,b). Hence, it is possible that our intervention did not reach the physical activity dose necessary to achieve beneficial effects on arousal and attention.

Other explanations for the discrepant findings might be the measurement used for behavioral self-regulation and the timing of the measurement. For example, Riley et al. (2016) reported increased time on-task during their physically active academic lessons based on observational data. Our measure, in contrast focused on behavior in the classroom, reported by the teachers at baseline and at follow-up. Hence, our measure expressed the behavior in the classroom over a long period in which, the majority of the academic lessons did not follow or include physical activity. Thus, it is possible that our intervention children were more on-task during the intervention activities compared to their control peers, as in Riley et al. (2016), but this was not captured by our way of measurement. Furthermore, the variance in the behavioral self-regulation factor observed across the intervention and control group teachers may have delimited the construct validity thus explaining the lack of intervention effect on this mediator. The use of teacher rated behavioral self-regulation is furthermore a subject to observer bias (McClelland and Cameron, 2012). Direct observation of behavioral self-regulation was out of scope for the present study.

The relation between the change in behavioral self-regulation and change in academic performance in numeracy is an important finding, emphasizing the significant role of behavioral self-regulation for academic performance also in an older age group (fifth-grade children) than previously shown (preschoolers and first-grade children) (von Suchodoletz et al., 2013; Gestsdottir et al., 2014; Schmitt et al., 2014).

### The Role of School Related Well-Being

Our intervention did not improve school related well-being. Moreover, change in school related well-being was not related to change in numeracy. This is in line with the quasi-experimental study by Kall et al. (2015), who did not observe any effects of a curricular physical activity intervention on any of the Kidscreen dimensions in elementary school children. An important limitation in the Kall et al. (2015) study, however, was that they did not include objectively measurement of physical activity. Hence, it is possible that both their findings and ours revealing lack of effect on school related well-being are attributable to an insufficient dose. As observed by Norris et al. (1992), duration and intensity of physical activity may be of importance to affect emotions.

Furthermore, it could also well be that our intervention activities and the mode of delivery did not stimulate positive peer-relationships, social identity, and belonging, which all are important for well-being (Bailey, 2016; Orkibi and Ronen, 2017). Indeed, both type of activities and mode of delivery influence intervention effects (Morgan et al., 2016). In terms of delivery, the ASK study pre-intervention workshops for teachers emphasized a mastery- and autonomy supportive learning environment which has been shown to facilitate need satisfaction, quality of motivation, and psychological well-being (Ryan and Moller, 2017). However, our study included a large number of intervention teachers with variability of experience and expertise in organizing and facilitating physical activity. Hence, it is possible that the settings for the implementation of the intervention activities were different across schools with some teachers not being able to implement the physical activities such that children’s well-being were facilitated. For example, although group activities creates opportunities for cooperation, and facilitates belonging and mastery (Ryan and Moller, 2017), the different emphasis on the competition element potentially initiated by some intervention teachers may have led to various experiences of the activities among the children of which some then may have experienced reduced rather than enhanced their well-being. Indeed, teachers’ ability to create a safe learning environment, as well as promoting good social relationship and acceptance of peers, has previously been shown to be of major importance for well-being in the classroom (Holfve-Sabel, 2014). Intervention teachers may not have been able to put this into effect outside the classroom.

In terms of intervention activities, the children are different in terms of social and physical-motor competence to participate. This requires tailored activities. It is possible that the group of children in the present study joined the physical activities with less social and physical competencies to their disposal for involvement in and mastery of the group activities. Hence, the possible benefits of these activities for school related well-being might have been counteracted. Indeed, according to self-determination theory, psychological well-being rests upon satisfaction of basic needs, including not only social relatedness, but satisfaction of the need for competence as well (Orkibi and Ronen, 2017; Ryan and Moller, 2017). Future studies would do well to examine the role of these psychological prerequisites when examining well-being as a psychosocial mediating mechanism. At this point then we cannot rule out the possibility that, consistent with previous findings (Bergh et al., 2012), our intervention generated unintended effects on this special group of children.

### Strengths and Limitations

The cluster randomized controlled design and the large sample size, together with the statistical approach used to examine our hypotheses, are strengths of the present study. In line with the recommendations by Valente and MacKinnon (2017), we estimated the mediating effect in our RCT with an ANCOVA model, which increases the precision of the treatment effect through the adjustment for baseline scores. We furthermore ran the ANCOVA model with a latent variable SEM approach, which enabled testing of the full ANCOVA model with three latent mediating factors simultaneously. The use of latent mediating factors exclude measurement error which enhance reliability and avoids underestimation of both the path between the predictor and mediator and the mediator and the outcome, and an overestimation of the direct link between the predictor and the outcome (Cole and Maxwell, 2003). Yet, it is possible that analyzing each dimension of executive function separately could have yielded other results, as previous studies have reported effects of physical activity on only one dimension of executive function and not others (Schmidt et al., 2015; Pesce et al., 2016). However, we did also analyze each dimension separately, but this approach did not change any findings. A further strength is the inclusion several mediators measured by different data sources (executive function tests, teacher reports, and self-report by the children).

The main aim of the ASK study was to determine the effect of physical activity on academic performance. The present study is based on a secondary analysis using a subsample of children, and was not *a priori* defined. Thus, our results should be interpreted with this limitation in mind.

## Conclusion

In conclusion, neither change in executive function, behavioral self-regulation, nor school related well-being mediated the effect of the ASK intervention on performance in numeracy in these children performing lowest in numeracy. This finding might primarily be attributed to the lack of effect of the intervention on the mediators generated by lack of sufficient physical activity dose. Future studies should investigate mediators between physical activity and academic performance using interventions with a sufficient physical activity, over a longer time-span, using several measurement time points. Such research will be critical to augment our understanding of mechanisms for the suggested effect of physical activity on schoolchildren’s academic performance.

## Author Contributions

KA conceived the idea for the paper together with YO, performed the data collection, analyzed the data, and wrote the manuscript draft. EA contributed in data analyses and drafted the manuscript. JA contributed in data collection and interpretation of results. AL contributed in data analyses. GR obtained funding for the study. VM contributed in data collection and drafted the manuscript. YO helped out in interpretation of the results and drafted the manuscript. All authors read, commented on, and approved the final manuscript.

## Conflict of Interest Statement

The authors declare that the research was conducted in the absence of any commercial or financial relationships that could be construed as a potential conflict of interest.

## Footnotes

1 www.askstudy.no

2 www.askstudy.no
