# Supplementary material for: Executive Function, Behavioral Self-Regulation, and School Related Well-Being Did Not Mediate the Effect of School-Based Physical Activity on Academic Performance in Numeracy in 10-Year-Old Children. The Active Smarter Kids (ASK) Study
Source: Front Psychol. 2018 Feb 28;9:245. doi: 10.3389/fpsyg.2018.00245 (PMC5835798; doi:10.3389/fpsyg.2018.00245)
Supplement: Supplementary file 1 [file Table_1.DOCX]

Supplement 1:

**Table S1.** Factor loadings (standardized) for the latent variables in the mediation model.

| **Latent variables** | **Baseline** |  | **Follow-up** |
| --- | --- | --- | --- |
| **Executive function**  **Stroop CW**  **WISC-IV b**  **TMT-b** | 0.628*  0.309*  0.496* |  | 0.650*  0.277*  0.468* |
| **Behavioral self-regulation**  **Parcel 1**  **Parcel 2**  **Parcel 3**  **Parcel 4**  **Parcel 5** | 0.901*  0.881*  0.892*  0.920*  0.909* |  | 0.905*  0.894*  0.889*  0.930*  0.913* |
| **School related well-being**  **Item 1**  **Item 2**  **Item 3**  **Item 4** | 0.746*  0.771*  0.630*  0.611* |  | 0.788*  0.815*  0.761*  0.703* |

Note. Stroop CW; Stroop Color Word; WISC-IV b = Wechsler Intelligence Scale for Children, fourth edition, backward digit span; TMT-b = Trail Making Test part B; * p < .001
